# Beratung für Angehörige von Menschen mit Demenz – Ergebnisse der qualitativen Evaluation eines Demenzstützpunktes

**DOI:** 10.1007/s00391-025-02458-w

**Published:** 2025-07-03

**Authors:** Karina Fasse, Sven Schwabe, Franziska A. Herbst

**Affiliations:** Institut für Allgemeinmedizin und Palliativmedizin, Medizinische Hochschule Hannover, Carl-Neuberg-Straße 1, 30625 Hannover, Deutschland

**Keywords:** Demenzberatung, Informelle Fürsorge, Pflegende Angehörige, Qualitative Forschungsmethoden, Semistrukturierte Interviews, Dementia counselling, Informal caregiving, Family caregivers, Qualitative research methods, Semistructured interviews

## Abstract

**Hintergrund:**

Zwei Drittel der Menschen mit einer Demenz in Deutschland werden von Angehörigen betreut. Der Demenzstützpunkt Ammerland und Umgebung bietet diesen Angehörigen eine Beratung zu pflegerischen und sozialen Fragestellungen.

**Ziel der Arbeit:**

Studienziel war, den Demenzstützpunkt Ammerland zu evaluieren, indem die Erwartungen, Erfahrungen und Wünsche von Angehörigen, bezogen auf den Demenzstützpunkt, erfasst werden.

**Material und Methoden:**

Leitfadengestützte Interviews wurden mit Angehörigen von Menschen mit Demenz, die das Beratungsangebot des Demenzstützpunktes in Anspruch genommen haben, geführt. Die 12 Einzelinterviews wurden telefonisch zwischen März und Dezember 2023 durchgeführt und wörtlich transkribiert. Die Inhalte aus den Interviews wurden nach der qualitativen Inhaltsanalyse nach Mayring in der Datenanalyse-Software MAXQDA12 zusammenfassend ausgewertet.

**Ergebnisse:**

Die Angehörigen berichteten eine hohe Zufriedenheit mit dem Beratungsangebot des Demenzstützpunktes. Sie erlebten durch die Beratung eine Entlastung in der Betreuung der Personen mit Demenz. Als positiv wahrgenommen wurden die gute Erreichbarkeit, die hohe fachliche Kompetenz und das empathische Auftreten der Versorgungskoordinatorinnen. Bezüglich der Weiterentwicklung des Demenzstützpunktes wünschten sich die Angehörigen ein präsenteres Auftreten des Demenzstützpunktes und regelmäßige Veranstaltungen, wie etwa Angehörigentreffen.

**Diskussion:**

Im Hinblick auf den demografischen Wandel und die prognostizierte Zunahme von Menschen mit Demenz sollten entsprechende Beratungsangebote ausgeweitet und bedarfsorientiert weiterentwickelt werden.

**Zusatzmaterial online:**

Zusätzliche Informationen sind in der Online-Version dieses Artikels (10.1007/s00391-025-02458-w) enthalten.

## Abstract (en)

**Background:**

Of the people with dementia in Germany, two thirds are cared for by relatives. The Dementia Support Center in Ammerland and the surrounding area offers these relatives counselling on nursing and social issues.

**Objective:**

The purpose of the study was to evaluate the Dementia Support Center in Ammerland by investigating the expectations, experiences and wishes of relatives in relation to the Dementia Support Center.

**Material and methods:**

Guided interviews were conducted with relatives of people suffering from dementia who made use of the counselling service of the Dementia Support Center. The 12 individual interviews were conducted by telephone between March and December 2023 and transcribed verbatim. The contents of the interviews were summarized using the qualitative content analysis according to Mayring in the data analysis software MAXQDA12.

**Results:**

The relatives reported a high level of satisfaction with the counselling offered by the Dementia Support Center. Through the counselling they experienced a relief in caring for people with dementia. The good accessibility, high professional competence and empathetic demeanor of the care coordinators were perceived as positive. With respect to the further development of the Dementia Support Center, the relatives wanted the center to be more present and have regular events, such as family meetings.

**Conclusion:**

In view of demographic changes and the predicted increase in people with dementia, appropriate counselling services should be expanded and further developed in line with the needs.

Zwei Drittel der Menschen in Deutschland, die an einer Demenz erkrankt sind, werden von ihren Angehörigen betreut. Der Demenzstützpunkt Ammerland und Umgebung berät Angehörige von Menschen mit Demenz zu pflegerischen und sozialen Fragen. In der vorliegenden Studie wurden Angehörige, die das Beratungsangebot des Demenzstützpunktes in Anspruch genommen haben, mittels leitfadengestützter Interviews befragt. Der vorliegende Beitrag präsentiert die Erwartungen, Erfahrungen und Wünsche der Angehörigen in Bezug auf den Demenzstützpunkt.

## Hintergrund

In Deutschland leben etwa 1,8 Mio. Menschen mit einer Demenz, von denen zwei Drittel im häuslichen Umfeld von Angehörigen betreut werden [1]. Diese Betreuung ist mit zahlreichen Belastungen verbunden [2]. Ein demenzspezifisches Versorgungsmanagement, welches auf die individuelle Krankheits- und Lebenssituation der erkrankten Person abgestimmt ist, kann die Lebensqualität der Patient*innen verbessern und die Belastungen der Angehörigen verringern [3]. Entsprechend ist ein Ziel der Nationalen Demenzstrategie, Menschen mit Demenz und ihre Angehörigen durch Beratungen zu unterstützen [4]. Der Demenzstützpunkt Ammerland und Umgebung (Träger: plexxon Management gGmbH) ist eine regionale Anlaufstelle zur Beratung von Menschen mit einer Demenz und ihren Angehörigen. Das Leitbild des Demenzstützpunktes orientiert sich an einem personenzentrierten Ansatz. Die Versorgungskoordinatorinnen des Stützpunktes haben eine Ausbildung im medizinischen Bereich (z. B. Gesundheits- und Krankenpflegerin) und teils eine spezifische Weiterbildung im Bereich Case Management. Sie beraten und informieren u. a. zur Inanspruchnahme von Sozialleistungen und Hilfsangeboten und der Organisation der wohnortnahen Versorgung. Der Demenzstützpunkt wurde am 01.01.2021 gegründet und betreut jährlich durchschnittlich 270 Beratungsfälle.

## Ziel

Ziel der vorliegenden Studie ist es, Erwartungen, Erfahrungen und Wünsche von Angehörigen, die durch den Demenzstützpunkt beraten wurden, zu erfassen. Im Fokus stehen dabei das Erleben der Angehörigen bezüglich des Beratungs- und Vermittlungsangebots, Erfahrungen mit den Unterstützungsangeboten und der Kommunikation sowie Wünsche zur Weiterentwicklung des Demenznetzwerkes.

## Methoden

### Studiendesign

Die Studie kam auf Initiative des Demenzstützpunktes zustande. Der Demenzstützpunkt hatte keinen Einfluss auf die Studiendurchführung und -auswertung. Das Studiendesign basiert auf einem qualitativ-explorativen Ansatz. Es wurden leitfadengestützte Interviews mit Angehörigen von Menschen mit einer Demenz, die das Beratungsangebot des Demenzstützpunktes in Anspruch genommen haben, geführt. Ergänzend wurden mit einem strukturierten Fragebogen soziodemografische Charakteristika der Teilnehmenden erfasst.

### Rekrutierung und Auswahl der Studienteilnehmenden

In der Studie befragt wurden sowohl (1) Angehörige, die ein erstmaliges Informationsgespräch mit der Versorgungskoordinatorin des Demenzstützpunktes in Anspruch genommen hatten (Erstkontakt) als auch (2) Angehörige, die bereits mehrfach von den Versorgungskoordinatorinnen beraten wurden (Folgekontakt). Dadurch sollten Erfahrungen aus unterschiedlichen Stadien des Beratungsprozesses abgebildet werden. Alle Teilnehmenden wurden zwischen März und Dezember 2023 im Rahmen der Beratungsgespräche als Gelegenheitsstichprobe bezüglich der Charakteristika „Erstkontakt“ und „Folgekontakt“ von den Versorgungskoordinatorinnen rekrutiert. Eingeschlossen wurden ausschließlich volljährige Angehörige mit für eine Studienteilnahme ausreichenden deutschen Sprachkenntnissen. Im Vorgespräch mit dem Team des Demenzstützpunktes wurde festgestellt, dass bisher keine Beratungsgespräche, in denen eine Sprachbarriere bestand, stattgefunden hatten.

### Leitfadenentwicklung und -aufbau

Gemeinsam mit dem Team des Demenzstützpunktes wurden in einem Online-Workshop relevante Evaluationsthemen identifiziert und nach der SPSS-Methode (Sammeln, Prüfen, Sortieren und Subsumieren der Fragen) in einen Leitfaden überführt [5]. Es wurden 2 Leitfadenversionen mit angepassten Fragen für die beiden Teilnehmendengruppen (Erst- bzw. Folgekontakt; s. Zusatzmaterial online: Anhänge 1 und 2) erstellt. Die Interviewleitfäden wurden hinsichtlich der genutzten Begriffe und Formulierungen getestet und bestätigt. Testpersonen waren eine Versorgungskoordinatorin und eine pflegende Angehörige, die über persönliche Kontakte rekrutiert wurde. Die Ergebnisse des Pretests sind nicht in die Datenauswertung eingegangen. Der Leitfaden bildet 5 Themenbereiche ab: (i) Erleben des Kontaktes zum Demenzstützpunkt, (ii) Erleben der Beratung durch die Versorgungskoordinatorin, (iii) Beratung zum Thema Palliativversorgung, (iv) Einbindung und Förderung des sozialen Umfeldes sowie (v) Weiterentwicklung des Demenzstützpunktes.

### Interviewdurchführung und -auswertung

Die Einzelinterviews wurden telefonisch von der Erstautorin (K.F.) durchgeführt. Vor Studienbeginn bestand keine Beziehung zwischen der Erstautorin und den Teilnehmenden. Die Interviews wurden audioaufgezeichnet, wörtlich transkribiert und dabei pseudonymisiert. Die Transkription erfolgte durch die Erstautorin und eine studentische Hilfskraft und wurde durch die Erstautorin überprüft. Die Inhalte aus den Interviews wurden nach der qualitativen Inhaltsanalyse nach Mayring mit der Datenanalysesoftware MAXQDA12 zusammenfassend ausgewertet [6]. Die Entwicklung des Kategoriensystems orientierte sich zunächst deduktiv an den Themen des Interviewleitfadens und wurde im weiteren Verlauf um Kategorien aus den Transkripten induktiv ergänzt. Alle Interviews wurden von der Erstautorin (K.F.) kodiert. Ein Interview wurde von einer weiteren Forscherin des Instituts (T.B.) unabhängig co-kodiert. Die beiden Forscherinnen konsentierten anschließend ihre Interviewkodierungen. Die Letztautorin (F.H.) führte eine Konsistenzprüfung aller kodierten Interviews durch. Nach 12 Interviews konnte eine relative Datensättigung verzeichnet werden, da neue Interviews keine neuen Inhalte zur Fragestellung lieferten. So wurde die Erhebung geschlossen.

## Ergebnisse

### Studienpopulation

Es konnten 12 Personen für die Studie rekrutiert werden; fünf waren männlich, 7 weiblich. Die Einzelinterviews dauerten zwischen 19 und 63 min. Sechs der 12 Interviewpartner*innen pflegten einen Elternteil und die anderen 6 ihre/n Ehepartner*in. Zum Zeitpunkt des Interviews hatten 5 Personen ein einmaliges Beratungsgespräch und 7 Person bereits mehrere Gespräche in Anspruch genommen. Das Durchschnittsalter der Interviewpartner*innen betrug 65 Jahre (SD ±16,1 Jahre) (Tab. 1).

**Tab. 1:** Soziodemografische Charakteristika der Studienteilnehmenden

Teilnehmende Angehörige (*n* = 12)	
*Interviewlänge in Minuten*	31,8; min. 19, max. 63
*Alter in Jahren*	65; min. 43, max. 92
*Geschlecht, n*	
Weiblich	7
Männlich	5
*In einer Partnerschaft lebend, n*	
Ja	11
Nein	1
*Geburtsland, n*	
Deutschland	11
Polen	1
*Beschäftigungsverhältnis, n*	
Vollzeitbeschäftigt	2
Teilzeitbeschäftigt	1
Nicht erwerbstätig	2
In Ruhestand	7
*Weitere Personenanzahl im Haushalt, n*	
0	1
1	5
2	2
3	2
4	1
Keine Angabe	1
*Weitere Personenanzahl unter 18-Jähriger im Haushalt, n*	
0	9
1	1
3	1
Keine Angabe	1
*Beziehung zur demenziell erkrankten Person, n*	
Ein Elternteil	6
Ehepartner/in	6
*Alter der demenziell erkrankten Person in Jahren*	79,5; min. 68, max. 88
*Geschlecht der demenziell erkrankten Person, n*	
Weiblich	8
Männlich	4
*Wohnsituation der demenziell erkrankten Person, n*	
Eigenes Zuhause	8
Pflegeheim	2
Beim im Interview befragten/r Angehörigen	2
*Weitere Personenanzahl im Haushalt der demenziell erkrankten Person, n*	
1	6
2	1
3	1
Nicht zutreffend	3
Keine Angabe	1
*Demenzdiagnose bereits erhalten, n*	
Ja	11
Im Diagnoseprozess	1
*Demenzform, n*	
Alzheimer-Demenz	3
Vaskuläre Demenz	1
Demenz bei M. Parkinson	1
Frontotempolare Demenz	1
Unbekannt	4
Aktuell im Diagnoseprozess	1
Keine Angabe	1
*Zeitlicher Abstand zwischen Diagnose und Interview in Jahren, n*	
0	3
1	2
2	4
9	1
Noch im Diagnostikprozess	1
Keine Angabe	1
*Folgetermine zum Zeitpunkt des Interviews stattgefunden, n*	
Nein, nur Erstkontakt	5
Ja, Folgekontakt(e)	7
Anzahl der Folgetermine, n*	
1	2
5	3
7	1
Keine Angabe	1

*Die Anzahl der Folgetermine wurde nur von 7 Teilnehmenden erhoben; die anderen 5 Teilnehmenden hatten zum Interviewzeitpunkt ein einmaliges Beratungsgespräch

### Ergebnisse der Codeanalyse der qualitativen Interviews

Insgesamt wurden 7 inhaltliche Kernkategorien herausgearbeitet: (1) Erwartungen, (2) Kontaktaufnahme, (3) Beratungsangebot, (4) soziales Umfeld des Angehörigen, (5) Lebensende und Palliativversorgung, (6) Weiterentwicklung des Demenzstützpunktes, (7) weitere Angebote des Demenzstützpunktes (s. Zusatzmaterial online: Anhang 3). Eine Tabelle mit Kodierbeispielen befindet sich im Zusatzmaterial online: Anhang 3.

#### Erwartungen

Die Befragten äußerten diverse Erwartungen an den Demenzstützpunkt vor ihrem ersten Beratungsgespräch. Sie erhofften sich Informationen zum Erkrankungsbild, eine Orientierung für die neue Lebenslage und eine Übersicht der Unterstützungsmöglichkeiten. Auch wünschten sie sich Unterstützung bei bürokratischen Aufgaben (z. B. zur Einstufung des Pflegegrades), Hinweise für den Umgang mit ihren an Demenz erkrankten Angehörigen und Informationen zum Austausch in einer Selbsthilfegruppe. Alle Angehörigen berichteten, dass ihre Erwartungen im Laufe der Beratungen erfüllt oder sogar übertroffen wurden.

#### Kontaktaufnahme

Die Angehörigen wurden durch eigenständige Internetrecherche, über Empfehlungen aus ihrem sozialen Umfeld, ein Pflegeservicebüro und Hausärzt*innen auf den Demenzstützpunkt aufmerksam.

Die Kontaktaufnahme zum Demenzstützpunkt erfolgte anlässlich der erstmaligen Diagnose der Erkrankung Demenz und der erlebten Überforderung in der häuslichen Betreuung und Pflege. Häufig lag bei der ersten Kontaktaufnahme zum Demenzstützpunkt bereits eine Demenzdiagnose vor. In anderen Fällen befanden sich die Betroffenen noch im Diagnostikprozess, oder die Diagnostik wurde erst durch die Unterstützung der Versorgungskoordinatorin eingeleitet.

Einige Angehörige hätten sich bereits zu einem früheren Zeitpunkt Kontakt zum Demenzstützpunkt gewünscht, allerdings war ihnen das Beratungsangebot nicht bekannt. Andere schätzten den Zeitpunkt der ersten Kontaktaufnahme als passend ein.

#### Beratungsangebot

Alle Studienteilnehmenden berichteten, dass die Beratungen durch die Versorgungskoordinatorinnen des Demenzstützpunktes ihren individuellen Bedürfnissen entsprochen haben. Die Versorgungkoordinatorinnen nahmen sich Zeit, um auf individuelle Probleme einzugehen, und konnten situationsabhängig vielseitige Unterstützungsangebote vermitteln: Tagespflege, Ergo- und Physiotherapie, Facharzttermine, ehrenamtliche Begleitpersonen und Haushaltshilfen. Die Angehörigen erhielten eine Übersicht über die passenden lokalen Unterstützungsangebote und teilweise Empfehlungen für einen passenden Dienstleister. Andere Angehörige berichteten, dass die Versorgungskoordinatorinnen Termine bei Dienstleistern für die Angehörigen vereinbarten.

Die Angehörigen nahmen die Beratungsgespräche als Entlastung war. Sie würden emotionale Bestärkung für die Betreuungsarbeit erfahren und Tipps für den sozialen Umgang mit der Person mit Demenz erhalten. Die Angehörigen erlebten sich dadurch als weniger ängstlich und alleingelassen und machten Selbstwirksamkeitserfahrungen: „Die Summe, dass da jemand ist, der kommt, der einem hilft in einer Notlage, aus der man selber nicht rauskommt. […] Die Angst lähmt einen auch, sie können gar nicht mehr klar denken. […] Dass da jemand ist, der einem da raushilft aus dieser Zwangslage, das ist das Entscheidende“ (A0040). Als wertvoll erlebten sie zudem die Unterstützung bei der Diagnosestellung, Informationen über die Demenzerkrankung und Unterstützung bei der Organisation der häuslichen Pflege. Sie profitierten von der gewonnenen Zeit und dem emotionalen Abstand durch die externe Betreuung sowie die Vermittlung der ehrenamtlichen Begleitpersonen und die vermittelte Haushaltshilfe. Unterstützung erfolgte zudem durch die Informationen zu finanziellen Entlastungsmöglichkeiten.

Die Erreichbarkeit der Versorgungskoordinatorinnen wurde positiv wahrgenommen. Einige Angehörige erhielten eine stets erreichbare Mobilfunknummer, was den Angehörigen Sicherheit vermittelte. Andere Angehörige erklärten, dass sie keine Notfallnummer erhielten und sich diese gewünscht hätten.

Nach den ersten Beratungsgesprächen unterschied sich das weitere Prozedere: In einigen Fällen wurde direkt ein Folgetermin mit der Versorgungskoordinatorin vereinbart, in anderen nicht. Nach der Erstberatung wollten einige selbst Kontakt aufnehmen, andere bevorzugten eine Kontaktaufnahme durch den Demenzstützpunkt nach 3 bis 6 Monaten.

Alle Befragten empfanden den Kontakt mit den Versorgungskoordinatorinnen positiv. Dabei wurde der freundliche, höfliche, entgegenkommende und empathische Umgang geschätzt: „Also nur positiv. Die waren alle sehr freundlich, sehr kompetent und ja höchst flexibel. Also wir haben nur positive Erfahrungen gemacht“ (A0047). Die Angehörigen empfanden die Versorgungskoordinatorinnen als kompetent und engagiert. Ihre Rolle wurde mitunter auch empfunden als die einer Mediatorin, die familiäre Konflikte entschärfen konnte. Der positive Eindruck verstärkte sich im Verlauf der Beratungsgespräche.

#### Soziales Umfeld der Angehörigen

Die Angehörigen gaben an, dass die Organisation der Betreuung ihres Angehörigen mit Demenz in der Tagespflege bewirkte, dass sie mehr Freizeit hatten, die sie z. B. für die Pflege eigener sozialer Kontakte nutzten. Manche benötigten keine Unterstützung bei der Organisation der eigenen Teilhabe am sozialen Leben.

Beratungsgespräche fanden in unterschiedlichen Konstellationen statt: allein mit der Versorgungskoordinatorin, mit dem Angehörigen mit Demenz und mit weiteren nahen Angehörigen. Die Gesprächskonstellationen wurden in allen Fällen als passend empfunden.

#### Lebensende und Palliativversorgung

Das Lebensende der Person mit Demenz und eine mögliche palliative Versorgung wurden in den Beratungsgesprächen nicht angesprochen. Dies empfanden alle Befragten als passend, da in ihrer Wahrnehmung das Thema Lebensende noch weit entfernt war und sie sich noch nicht damit beschäftigen wollten. Die Angehörigen gaben an, dass sie sich bei Bedarf bei den Versorgungskoordinatorinnen melden würden.

#### Weiterentwicklung

Die Angehörigen würden den Demenzstützpunkt grundsätzlich weiterempfehlen: „Wenn ich in einem Gespräch wäre mit Freunden, Bekannten […] ich habe den Demenzstützpunkt schon weiterempfohlen, fällt mir gerade ein. Also ich würde ihn auf jeden Fall weiterempfehlen. Mit ganz vielen Ausrufezeichen“ (A0054). Zugleich kommunizieren die Angehörigen diverse Ideen zur Weiterentwicklung. Der Demenzstützpunkt solle deutlich präsenter werden, damit Betroffene die Möglichkeit hätten, frühzeitig den Kontakt zu suchen. Manchmal kämen Empfehlungen von Ärzt*innen zu spät; dann solle es die Möglichkeit geben, unabhängig von dem/der Ärzt*in mit dem Demenzstützpunkt in Kontakt zu treten. Wünschenswert wäre, wenn direkt bei einer Demenzdiagnose auf den Demenzstützpunkt hingewiesen würde. Zum Teil nahmen Angehörige eine Überlastung des Personals des Demenzstützpunktes wahr und wünschten sich eine personelle Aufstockung des Stützpunktes.

Einige Angehörige befürworteten das Angebot von Sprechstunden in öffentlichen Räumen, da sie die Umgebung abseits der eigene Häuslichkeit schätzten. Andere bevorzugten die Beratung zu Hause, da sie ihre Angehörigen mit Demenz nicht allein lassen könnten.

#### Weitere Angebote des Demenzstützpunktes

Einige Angehörige berichteten, dass sie „Vergissmeinnicht“-Boxen von Hausärzt*innen bei Feststellung der Demenzerkrankung erhielten. Die Boxen enthalten erste Informationen und die Kontaktdaten des Demenzstützpunktes und wurden als sehr hilfreich beschrieben.

Einige Angehörige schätzten, dass sie die gesetzlich vorgeschriebenen Pflegeberatungen zum Erhalt des Pflegegrades ihrer Angehörigen mit einer Demenz mit den Versorgungskoordinatorinnen des Demenzstützpunktes durchführen könnten: „Für den Pflegegrad muss ja halbjährlich jemand zur Beratung kommen und gucken, wie es läuft. Da hat sie gesagt, das könnten die auch übernehmen. Also da musste ich mir nicht […] irgendjemand Fremdes […suchen]. Ich bin auch ein bisschen froh, dass das alles so ein bisschen zentral dort geregelt wird“ (A0042).

Der Demenzstützpunkt hat in der Vergangenheit Angehörigentreffen und Workshops zu verschiedenen die Demenz betreffenden Themen organisiert; diese waren einigen Angehörigen jedoch nicht bekannt. Hier wünschten die Angehörigen sich eine stärkere Bewerbung. Andere äußerten aktuell keinen Bedarf an entsprechenden Veranstaltungen, da sie mit der Betreuung für die Person mit einer Demenz beschäftigt seien. Einige Angehörige berichteten, dass sie an einmaligen Angehörigentreffen oder Workshops teilgenommen haben und sich regelmäßigere Veranstaltungen wünschen würden, wie Angehörigentreffen und Workshops, um sich zum Thema Demenz fortzubilden. Voraussetzung für die Teilnahme an solchen Angeboten sei aber die Betreuung ihres Angehörigen mit Demenz während der Veranstaltungszeit.

## Diskussion

Ziel der Studie war, den Demenzstützpunkt Ammerland zu evaluieren, indem Erfahrungen und Wünsche der durch den Demenzstützpunkt betreuten Angehörigen erfasst wurden. Bisher ist den Autor*innen keine Studie bekannt, die Perspektiven der Angehörigen von Menschen mit Demenz auf Demenzberatungen untersucht [7].

Die befragten Angehörigen waren mit dem Beratungsangebot des Demenzstützpunktes durchweg sehr zufrieden und erlebten durch die Beratung vielfältige Entlastungen in der Betreuung der Personen mit Demenz. Die Betreuung einer Person mit Demenz bedeutet für Angehörige häufig eine große Belastung [2, 8]. Indem der Demenzstützpunkt Ammerland Beratungen zur Inanspruchnahme von Sozialleistungen und Hilfsangeboten sowie die Koordinierung der wohnortnahen Versorgung der Person mit Demenz anbietet, kann er pflegende Angehörigen bei dieser Aufgabe unterstützen [2]. Entsprechend bedient der Demenzstützpunkt große Teile des Handlungsfelds 2 „Menschen mit Demenz und ihre Angehörigen unterstützen“ der Nationalen Demenzstrategie [4]. Die Studienteilnehmenden lobten die gute Erreichbarkeit, die hohe fachliche Kompetenz und das empathische Auftreten der Versorgungskoordinatorinnen. Zur Weiterentwicklung des Demenzstützpunktes wünschten sich die Angehörigen eine größere Sichtbarkeit des Demenzstützpunktes und regelmäßige Veranstaltungen, wie Angehörigentreffen und Workshops, um sich zum Thema Demenz fortzubilden. Voraussetzung, um an Veranstaltungen des Demenzstützpunktes teilzunehmen, sei allerdings eine adäquate Betreuung der Person mit einer Demenz in der Zeit der Veranstaltung.

Möglichkeiten zur Inanspruchnahme von Schulungen für Angehörige zum Thema Pflege und Demenz sollten laut den Angehörigen als auch entsprechend des Maßnahmenkatalogs der Nationalen Demenzstrategie [4] ausgebaut werden.

Laut der S3-Leitlinie Demenzen der Deutschen Gesellschaft für Neurologie e. V. (DGN) und der Deutschen Gesellschaft für Psychiatrie und Psychotherapie, Psychosomatik und Nervenheilkunde e. V. (DGPPN) [9] sollen Ärzt*innen Angehörige von Menschen mit einer Demenz adressieren und deren offene Versorgungsbedürfnisse erkennen und ggf. die Angehörigen an Demenzberatungen weiterleiten [10].

**Abb. 1:**
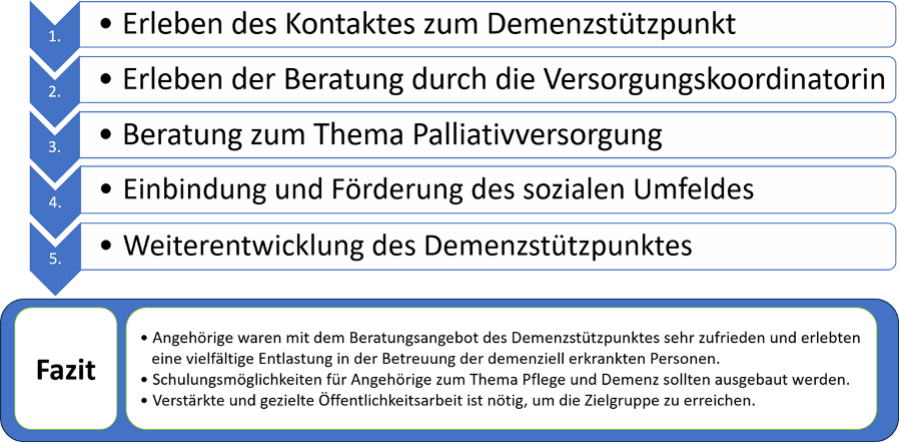
Graphical Abstract: Themenbereiche der Interviews und das resultierende Fazit der Interviewauswertung

Der Demenzstützpunkt arbeitet bereits mit einem großen Netzwerk von 185 ambulanten Ärzt*innen verschiedener Fachrichtungen zusammen, um auf das Angebot aufmerksam zu machen. Allerdings berichten Angehörige z. T., dass sie zu spät von dem Beratungsangebot erfahren hätten. Diese Erfahrung deckt sich mit anderen Studien, deren Autoren ebenfalls berichten, dass Ärzt*innen in der Mehrheit den Beratungsbedarf von Angehörigen mit Demenz nicht erkennen [11]. Somit sollte der Demenzstützpunkt über sein Netzwerk weitere Möglichkeiten suchen, um auf sich und seine Veranstaltungen aufmerksam zu machen. Mögliche Ideen dazu wären, aktuelle Veranstaltungshinweise in der Lokalpresse oder über lokale Radiosender anzukündigen. Angehörige, die bereits das Beratungsangebot in Anspruch genommen haben, könnten sich zudem für einen Newsletter registrieren lassen. Die „Vergissmeinnicht“-Boxen, die erste Informationen und die Kontaktdaten des Demenzstützpunktes enthalten, wurden als sehr positiv bewertet und sollten weiterhin genutzt werden. Die Angehörigen gaben teilweise sehr unterschiedliche Erwartungen und Präferenzen bezüglich der Beratungsgespräche an, sodass den Versorgungskoordinator*innen ein hohes Maß an Individualität abverlangt wird, um diesen zu entsprechen. Die heterogenen Erwartungen bei der Begleitung und Beratung wurden auch in einer Studie zum Versorgungsmanagement in Deutschland erhoben [12]. Die fachliche und soziale Kompetenz der Versorgungskoordinator*innen ist wesentlich für die Zufriedenheit der Angehörigen. Daher ist die Schulung der Versorgungskoordinator*innen wichtig (s. Abb. 1 zu Themenbereichen der Interviews und daraus resultierendem Fazit der Interviewauswertung).

Durch den demografischen Wandel gewinnt die Versorgung von Menschen mit Demenz erheblich an gesellschaftlicher Relevanz. Für das Jahr 2050 wurde eine Prognose von 2,8 Mio. Menschen mit einer Demenz in Deutschland errechnet [13]. Im Hinblick auf den demografischen Wandel, den Pflegekräftemangel und den Wunsch der Angehörigen nach regelmäßigen Veranstaltungen abseits der Beratungen, wäre ein Ausbau von Demenzstützpunkten sinnvoll.

### Limitationen

- Die Rekrutierung der Angehörigen erfolgte durch die Versorgungskoordinatorinnen des Demenzstützpunktes. Es kann nicht ausgeschlossen werden, dass Angehörige, die die Beratung als positiv erlebt haben, vorzugsweise rekrutiert wurden.
- Die Forschungsergebnisse sind aufgrund der besonderen Struktur und Finanzierung des Demenzstützpunktes Ammerland und Umgebung nur bedingt auf andere Beratungsinstitutionen zum Thema Demenz übertragbar.

## Ausblick

Die vorliegenden Studienergebnisse liefern erste Anhaltspunkte dafür, wie der Demenzstützpunkt Ammerland und andere Institutionen, die Demenzberatungen anbieten, ihr Angebot für die Angehörigen ausbauen könnten. Weitere Forschung zu der Perspektive der Versorgungskoordinator*innen und den Menschen mit einer Demenz ist notwendig, um das Weiterentwicklungspotenzial des Demenzstützpunktes aus ihrer Sicht zu erheben. Andere Anliegen des Demenstützpunktes, wie die Förderung der Stärkung der gesellschaftlichen Teilhabe von Menschen mit Demenz, sollten evaluiert werden, um zu erfassen inwieweit dieses Ziel erreicht wird.

## Fazit für die Praxis

- Angehörige waren mit dem Beratungsangebot des Demenzstützpunktes sehr zufrieden und erlebten vielfältige Entlastungen in der Betreuung der demenzerkrankten Personen.
- Schulungsmöglichkeiten für Angehörige zum Thema Pflege und Demenz sollten ausgebaut werden.
- Verstärkte und gezielte Öffentlichkeitsarbeit ist nötig, um die Zielgruppe zu erreichen.

## Supplementary Information

Anhang 1: Semistrukturierter Interviewleitfaden für Erstkontakte

Anhang 2: Semistrukturierter Interviewleitfaden für Folgekontakte

Anhang 3: Kodierbeispiele

## Data Availability

Die Rohdaten zu dieser Veröffentlichung können aufgrund geltender Beschränkungen nicht öffentlich zugänglich gemacht werden. Die Daten enthalten die Transkriptionen von Interviews. Keiner der Befragten hat der Weitergabe der vollständigen Transkription seines Interviews zugestimmt.
