# Supplementary material for: Consulting service for family members of people with dementia—Results of the qualitative evaluation of a dementia support center
Source: Z Gerontol Geriatr. 2025 Jul 3;59(1):38–44. [Article in German] doi: 10.1007/s00391-025-02458-w (PMC12823747; doi:10.1007/s00391-025-02458-w)
Supplement: Supplementary file 1 — Anhang 1: Semistrukturierter Interviewleitfaden für Erstkontakte [file 391_2025_2458_MOESM1_ESM.docx]

| **OFFENE ERZÄHLAUFFORDERUNG** | **OPTIONALE THEMENÜBERGREIFENDE FRAGEN** |
| --- | --- |
| Gerne möchte ich heute mit Ihnen auf Ihre Beratungsgespräche im Demenzstützpunkt Ammerland zurückblicken. Möchten Sie einfach mal berichten, wie Sie vom Demenzstützpunkt erfahren haben und was Sie bewegt hat Kontakt zum Demenzstützpunkt aufzunehmen? | **Details, Gründe, Erklärungen**  Bitte beschreiben Sie das näher!  Was ist ein Beispiel dafür?  Woran machen Sie das fest?  Wie erklären Sie sich das?  Weshalb ausgerechnet… (diese)?  Wie hat sich das dahin entwickelt?  Was bedeutet das für Sie?  **Erleben**  Wie erleben Sie das?  Wie fühlt sich das an?  Wie empfinden Sie das persönlich?  **Erwartungen, Wünsche**  Was erwarten/erhoffen Sie sich davon?  Was wünschen Sie sich (bezogen auf…)?  Was würden Sie gerne verändern? |
| **KERNFRAGEN** |  |
| **Erleben des Erstkontakts** |  |
| Weshalb haben Sie genau zu diesem Zeitpunkt Kontakt zum Demenzstützpunkt aufgenommen?  Inwiefern war Ihnen zu diesem Zeitpunkt die Demenzerkrankung ihres Angehörigen bekannt?  Inwiefern war dieser Zeitpunkt der ersten Kontaktaufnahme für Sie passend?--- Würden Sie dies rückblickend anders bewerten?---- Falls ja: Was hat Ihnen die Kontaktaufnahme erleichtert?  Welche Erwartungen hatten Sie an den Demenzstützpunkt? |  |
| **Erleben der Beratung** |  |
| Wie haben Sie den Kontakt zum Demenzstützpunkt erlebt?  Warum kam es nicht zu einer weiteren Beratung durch den Demenzstützpunkt?  Was hat sich für Sie durch die Beratung des Demenzstützpunkt verbessert? Inwiefern haben Sie Entlastung durch das Beratungsangebot erfahren?  Wie sind Ihre Erfahrungen mit den vermittelten Unterstützungsangeboten des Netzwerkes des Demenzstützpunktes?  Inwieweit ist in der Beratung auf Ihre individuelle Bedürfnisse eingegangen worden? |  |
| **Palliative Versorgung ( nur bei Bedarf)** |  |
| Inwiefern wurde in der Beratung das Lebensende der demenzerkrankten Person angesprochen?  Inwiefern hat sich durch die Auseinandersetzung mit dem Thema Lebensende etwas verändert?  Inwieweit wurde in der Beratung über eine hospizlich palliative Versorgung gesprochen? |  |
| **soziale Umfeld** |  |
| Inwieweit nehmen außer Ihnen noch weitere Ihrer Angehörigen an den Angeboten des Demenzstützpunktes teil?  Inwiefern wünschen Sie sich, dass weitere Angehörige in der Beratung miteinbezogen werden?  Inwieweit erleben Sie den Demenzstützpunkt als Unterstützung für Ihr soziales Umfeld? |  |
| **Weiterentwicklung** |  |
| Bisher haben wir viel über Ihre Erfahrungen mit dem Demenzstützpunkt geredet, nun würde ich gerne mit Ihnen über Weiterentwicklungsmöglichkeiten des Demenzstützpunktes sprechen.  **Erstkontakt/ Kommunikation**  Wie sollte Ihrer Meinung nach, nach dem Erstkontakt weiterverfahren werden?  Inwiefern wünschen sie sich, dass der Demenzstützpunkt nach der ersten Beratung aktiv Kontakt zu Ihnen aufnimmt?--- Nach welchem Zeitraum würden Sie eine Kontaktaufnahme als sinnvoll erachten?  **Erreichbarkeit**  Inwiefern würden Sie das Beratungsangebot des Demenzstützpunktes in öffentlichen Räumen (z.B. Sprechstunden im Rathaus oder im Gemeindezentrum) nutzen?  **Angebote** Zu Beginn des Gespräches haben Sie Ihre Erwartungen an den Demenzstützpunkt genannt. Diese waren … Inwiefern wurden Ihre Erwartungen an den Demenzstützpunkt erfüllt?  Welche Wünsche für die Weiterentwicklung des Demenzstützpunktes haben Sie? |  |
| **Abschluss** |  |
| Inwiefern würden Sie den Demenzstützpunkt weiterempfehlen? Gibt es bezogen auf die Inanspruchnahme an des Demenzstützpunktes Ammerland etwas, das für Sie wichtig ist und wonach ich Sie noch nicht gefragt habe?  Haben Sie noch Fragen an mich?  *Ich danke Ihnen herzlich für das Gespräch.* | |
