# Supplementary material for: Consulting service for family members of people with dementia—Results of the qualitative evaluation of a dementia support center
Source: Z Gerontol Geriatr. 2025 Jul 3;59(1):38–44. [Article in German] doi: 10.1007/s00391-025-02458-w (PMC12823747; doi:10.1007/s00391-025-02458-w)
Supplement: Supplementary file 3 — Anhang 3: Kodierbeispiele [file 391_2025_2458_MOESM3_ESM.docx]

| **Kernkategorie** | **Kode** | **Ankerbeispiel** |
| --- | --- | --- |
| Kontaktaufnahme | Wie aufmerksam geworden auf den Demenzstützpunkt | „Die Hausärztin, die habe ich versucht um Hilfe zu bitten, was nicht so besonders gut geklappt hat, aber die haben mir immerhin die Frau A [meint: Versorgungskoordinatorin] vermittelt. Fantastisch. […] Ich glaube die haben mir sogar den Informationskarton mitgegeben.“ (A0042) |
|  | Demenzerkrankung bekannt | „Also die Demenzerkrankung von meinem Vater war ja schon länger bekannt.“ (A0038) |
|  | Erwartungen | „Darlegung von Hilfsangeboten, Beratung […] erstmal im Vorfeld. Wo kann ich welche Hilfe bekommen? Wo sind welche Institutionen? Wo sind welche Wohngemeinschaften? Wo gibt es geschlossene Einrichtungen? Falls ich meinen Mann nicht mit nach Hause nehmen kann.“ (A0036) |
|  | Bewertung des Zeitpunktes der Kontaktaufnahme | „Also ich wäre froh gewesen, wenn ich den Demenzstützpunkt viel früher im Boot gehabt hätte.“ (A0042) |
|  | Zeitpunkt/Auslöser | „Der Zeitpunkt war einfach so weit fortgeschritten, dass ich mit meiner Mutter nicht mehr zurechtkam.“ (A0038) |
| Beratungsangebot | Individualität der Beratung | „Also was ich mit Frau A [meint: Versorgungskoordinatorin] hier an Sitzungen hatte war ganz auf mich zugeschnitten und unsere Probleme. Und ich hatte schon den Eindruck, dass sie sich vorbereitet hatte und wirklich im Fall drin war.“ (A0040) |
|  | Erfahrung mit vermittelten Unterstützungsangeboten | „Ganz toll, also da ist auch meine Mutter wirklich begeistert, weil die Menschen, die Pfleger, […] das Team dort, ist super lieb.“ (A0042) |
|  | Vermittlung von Unterstützungsangeboten | „Und sie hat also empfohlen, dass man einen Aufenthalt zur Diagnostik in der Klinik machen sollte. Sie hatte eine Klinik in Ort F [meint: einen Ort in der Region Ammerland] empfohlen und hat sich auch um einen Termin bemüht.“ (A0041) |
|  | Entlastung durch Beratung | „Und die Tagespflege das wäre auch nicht nötig, aber es ist für mich eine Entlastung, weil ich einen Tag dann Dinge erledigen kann.“ (A0044) |
|  | Verbesserung durch Beratung | „Die Summe, dass da jemand ist, der kommt, der einem hilft in einer Notlage aus der man selber nicht rauskommt. […] Die Angst lähmt einen auch, sie können gar nicht mehr klar denken. […] Dass da jemand ist, der einem da raushilft aus dieser Zwangslage, das ist das Entscheidende.“ (A0040) |
|  | Bewertung der Erreichbarkeit des Demenzstützpunktes | „Also so gesehen war die Erreichbarkeit immer gut gegeben. Und ich habe auch eine Handynummer unter der ich Person A [meint: Versorgungskoordinatorin] jederzeit erreiche.“ (A0038) |
|  | Bewertung des Kontaktes zum Demenzstützpunkt | „Also nur positiv. Die waren alle sehr freundlich, sehr kompetent und ja höchst flexibel. Also wir haben nur positive Erfahrungen gemacht.“ (A0047) |
|  | Weiteres Vorgehen nach Erstkontakt | „Wir haben halt immer Folgetermine vereinbart gleich.“ (A0047) |
| Lebensende und Palliativversorgung | Thematisierung der Palliativversorgung | „Das Einzige was wir geklärt haben ist, dass mein Mann auch hier zu Hause bis zum Ende sein kann. Vorausgesetzt ich kann das leisten.“ (A0036) |
|  | Ansprechen des Themas Lebensende in der Beratung | „Das ist völlig okay, dass das noch nicht stattfand, weil es geht meiner Mutter ja auch wieder wirklich recht gut und ich bin mir sicher, wenn es Thema wird, dass die Frau A [meint: Versorgungskoordinatorin] mich dann auch unterstützt.“ (A0042) |
| Soziales Umfeld Angehörige*r und Patient*in | Unterstützung für soziales Leben des Angehörigen + Patienten (Teilhabe) | „Das habe ich alles soweit privat schon organisiert. [So] dass ich im Moment eigentlich keinen weiteren Bedarf habe.“ (A0036) |
|  | Unterstützung für soziales Leben anderer Angehörige + Freunde | „Ich sehe durchaus ja Menschen auch in meinem Umfeld, Elternkreis meines Umfeldes, wo Demenz vorliegt, wo man sagen kann ‚Holt Euch mal Eure Hilfe da. Das macht schon Sinn.‘“ (A0057) |
|  | Wunsch, dass weitere Angehörige in Beratung einbezogen werden | „Nein. Das liegt auch daran, dass das bei uns nicht in Ort A [meint: Wohnort weiterer Angehöriger in Süddeutschland …] in der praktischen Versorgung gar nicht mit eingebunden ist.“ (A0036) |
|  | Weitere Angehörige in der Beratung | „Also mein Mann war immer dabei, und auch meine Tochter war mit dabei beim ersten Mal, mit der mein Mann ein gutes Verhältnis hat.“ (A0041) |
| Weiterentwicklung des Demenzstützpunktes | Gewünschtes Vorgehen nach Erstkontakt | „Für mich ist das so jetzt erstmal okay und wenn ich wieder Bedarf habe, dann würde ich mich lieber melden.“ (A0061) |
|  | Weiterempfehlung | „Also wenn ich in einem Gespräch wäre mit Freunden, Bekannten […] ich habe den Demenzstützpunkt schon weiterempfohlen, fällt mir gerade ein. Also ich würde ihn auf jeden Fall weiterempfehlen. Mit ganz vielen Ausrufezeichen.“ (A0054) |
|  | Wünsche für Weiterentwicklung | „Dass sie präsenter werden, damit man nicht über den Hausarzt davon erfährt, wenn es schon zu spät ist quasi.“ (A0042) |
|  | Erfüllung der Erwartungen der Angehörigen | „Das wurde alles so komplett erfüllt, wie ich mir das vorgestellt habe.“ (A0061) |
|  | Angebot in öffentlichen Räumen | „Mir ist es lieber Person A [meint: Versorgungskoordinatorin] kommt hierher, weil, denn muss ich keinen Ersatz [meint: Betreuungsperson für die Demenzerkrankte Person] suchen.“ (A0036) |
| Weitere Angebote des Demenzstützpunktes | Angehörigentreffen/Selbsthilfegruppe | „Ich sage mal im Moment ist es schwierig, weil unser Vater ja noch Zuhause lebt, der braucht ja auch eine Rundumbetreuung. Dann ist das schon schwierig. Aber ich sage mal, wenn es gar nicht geht, klar würde man das irgendwann vielleicht mal in Anspruch nehmen, wenn es erforderlich ist.“ (A0055) |
|  | Notfallbox | „Das war total toll, weil das kam tatsächlich auch zum Tragen, weil irgendwann ist meine Mutter gestürzt und der Notdienst wusste sofort Bescheid über alles.“ (A0047) |
|  | Pflegeberatung | „Für den Pflegegrad muss ja halbjährlich jemand zur Beratung kommen und gucken, wie es läuft. Da hat sie gesagt, das könnten die auch übernehmen. Also da musste ich mir nicht auch nicht noch extra irgendjemand Fremdes oder jemand Anderes suchen. Also ich bin schon, ich bin auch ein bisschen froh, dass das alles so ein bisschen zentral dort geregelt wird.“ (A0042) |
|  | Workshop | „Also ich wüsste nicht, dass solche Angebote [meint: Workshops zur Informationsvermittlung] da wären. Die gibt es nicht.  Wenn ja, dann würde ich teilnehmen, natürlich. Vielleicht ist meine Information auch nicht vollständig, das kann auch sein.“ (A0044) |
